# Genomic Comparison of the Closely-Related *Salmonella enterica* Serovars Enteritidis, Dublin and Gallinarum

**DOI:** 10.1371/journal.pone.0126883

**Published:** 2015-06-03

**Authors:** T. David Matthews, Robert Schmieder, Genivaldo G. Z. Silva, Julia Busch, Noriko Cassman, Bas E. Dutilh, Dawn Green, Brian Matlock, Brian Heffernan, Gary J. Olsen, Leigh Farris Hanna, Dieter M. Schifferli, Stanley Maloy, Elizabeth A. Dinsdale, Robert A. Edwards

**Affiliations:** 1 Department of Biology, San Diego State University, San Diego, California, 92182, United States of America; 2 Department of Computer Science, San Diego State University, San Diego, California, 92182, United States of America; 3 Computational Science Research Center, San Diego State University, San Diego, California, 92182, United States of America; 4 Theoretical Biology and Bioinformatics, Utrecht University, Utrecht, The Netherlands; 5 Centre for Molecular and Biomolecular Informatics, Radboud Institute for Molecular Life Sciences, Radboud University Medical Centre, Nijmegen, The Netherlands; 6 Department of Marine Biology, Institute of Biology, Federal University of Rio de Janeiro, Rio de Janeiro, Brazil; 7 Department of Microbiology, University of Illinois at Urbana-Champaign, Urbana, Illinois, United States of America; 8 Molecular Sciences Department, University of Tennessee Health Sciences Center, 858 Madison Ave, Memphis, Tennessee, United States of America; 9 University of Pennsylvania School of Veterinary Medicine, 3800 Spruce St, Philadelphia, Pennsylvania, 19104, United States of America; 10 Argonne National Laboratory, 9700 S. Cass Ave, Argonne, Illinois, 60349, United States of America; Institut National de la Recherche Agronomique, FRANCE

## Abstract

The *Salmonella enterica* serovars Enteritidis, Dublin, and Gallinarum are closely related but differ in virulence and host range. To identify the genetic elements responsible for these differences and to better understand how these serovars are evolving, we sequenced the genomes of Enteritidis strain LK5 and Dublin strain SARB12 and compared these genomes to the publicly available Enteritidis P125109, Dublin CT 02021853 and Dublin SD3246 genome sequences. We also compared the publicly available Gallinarum genome sequences from biotype Gallinarum 287/91 and Pullorum RKS5078. Using bioinformatic approaches, we identified single nucleotide polymorphisms, insertions, deletions, and differences in prophage and pseudogene content between strains belonging to the same serovar. Through our analysis we also identified several prophage cargo genes and pseudogenes that affect virulence and may contribute to a host-specific, systemic lifestyle. These results strongly argue that the Enteritidis, Dublin and Gallinarum serovars of *Salmonella enterica* evolve by acquiring new genes through horizontal gene transfer, followed by the formation of pseudogenes. The loss of genes necessary for a gastrointestinal lifestyle ultimately leads to a systemic lifestyle and niche exclusion in the host-specific serovars.

## Introduction

*Salmonella enterica* is a bacterial pathogen of reptiles, birds, and mammals, including humans [1]. There are currently ~2,600 recognized serovars [2], which cause a spectrum of diseases depending on the serovar and the host. While most *Salmonella* serovars have a broad host range, a small number are host-specific. Host-specific serovars are capable of infecting and causing disease in only one or a few closely related host species; for example, the causative agent of enteric, or typhoid fever is the human-specific serovar Typhi. The most common disease in humans caused by *Salmonella* is food-borne salmonellosis, a self-resolving gastroenteritis. Approximately 40,000 cases of food-borne salmonellosis are reported annually in the United States, but the estimated number of cases is 1.4 million as most cases are not diagnosed and reported [3,4]. While many different serovars have been implicated in outbreaks of food-borne salmonellosis in recent years, serovar Enteritidis associated with chickens is the second leading cause of food-borne salmonellosis in the United States [5]. An outbreak linked to Enteritidis-contaminated eggs occurred in the United States during the spring/summer of 2010 and was likely responsible for over 1,800 illnesses (http://www.cdc.gov/salmonella/enteritidis/index.html).

The first *Salmonella* genomes sequenced were from the serovar Typhimurium laboratory strain LT2 [6], and the multi-drug resistant serovar Typhi strain CT18 [7]. Subsequently, the genome of serovar Typhi strain Ty2 was sequenced, which allowed the first direct genomic comparison of *Salmonella* strains belonging to the same serovar [8]. While Ty2 and CT18 share more than 98% of genome sequence, there are numerous differences that distinguish them such as chromosomal rearrangements, and variation in their respective repertoires of prophages, pathogenicity islands and pseudogene content. A subsequent comparison of the genomes of serovar Typhimurium strains LT2 and 14028 showed differences in prophage and pseudogene content as well as dissimilar relative base substitution frequencies due to domestication of LT2, and gene polymorphisms that may explain the difference in virulence between these two strains [9]. The genomes of strains belonging to serovars Enteritidis and Gallinarum, a related avian-specific serovar, were compared to each other and the Typhimurium LT2 genome [10]. The Gallinarum genome was annotated with 309 putative pseudogenes; about three times more than the number of pseudogenes annotated in the Enteritidis genome and about one hundred more than the number of pseudogenes annotated in the similarly host-restricted serovar Typhi. The genomes of host-specific *Salmonella* serovars contain more pseudogenes than the broad host range serovars. These pseudogenes affect a variety of physiological functions including virulence, metabolism, and motility in serovar Gallinarum. Pseudogenes become fixed within host-restricted *Salmonella* because either there is less selective pressure to maintain those functions in the restricted niche, or the loss of function is selected for within the host. Pseudogene accumulation within a genome contributes to niche exclusion by limiting the genetic potential of the restricted organism.

Serovars Enteritidis, Dublin, and Gallinarum are closely related, with Dublin and Gallinarum diverging independently from an Enteritidis-like ancestor [10–13]. In spite of their close relationship, these serovars differ in host range and the diseases they cause. For example, serovar Dublin is a host-adapted serovar; its host range falls in between the broad host range of serovar Enteritidis and the host-restricted serovar Gallinarum. Serovar Dublin is adapted to cattle and causes an enteric fever, but can still infect multiple animal species including other domesticated animals and humans. As most infected humans also have an underlying medical issue or are immunocompromised, these infections often lead to a life-threatening bacteremia [14].

Serovar Gallinarum consists of two biotypes, Gallinarum and Pullorum, which cause two distinct disease states in fowl: fowl typhoid and pullorum disease respectively [15,16]. Serovar Gallinarum competitively excludes serovar Enteritidis from fowl by generating cross-immunity (the two serovars share the same immunodominant O-antigen) [17]. It is hypothesized that eradication of serovar Gallinarum from domestic fowl in the United States and England during the mid-20^th^ century opened up an ecological niche that serovar Enteritidis filled [18]. Since serovar Enteritidis is usually asymptomatic in chickens [19], contaminated eggs have entered the human food supply and cause the current outbreaks of Enteritidis-associated salmonellosis. In addition to their economic and public health importance, the different disease states and host ranges of these closely related serovars make them a good model system for studying the genetic basis of these traits.

In previous studies, the genome sequences of serovars Enteritidis and Gallinarum were compared [10], as well as the gene content of serovar Dublin relative to Enteritidis and Gallinarum [12] and the gene content of several Enteritidis strains using microarrays [20]. Recently Betancor *et al*. used a microarray to compare the gene content of 29 of these Enteritidis strains to a set of 4 Dublin strains [21]. Here we describe the genome sequences of Dublin SARB12 [22], a strain isolated from cattle, and Enteritidis LK5 [23], a strain derived from an isolate from chicken egg yolks obtained during a salmonellosis outbreak investigation, and compare these sequences to the published Dublin CT 02021853 (24), SD3246 [24] and Enteritidis P125109 [10] genome sequences. In addition, we compare the published Gallinarum genome sequence [10] to a recently sequenced biotype Pullorum strain. Through these comparisons we identify the genetic differences between these strains, and clarify the differences responsible for the varied host range and spectrum of disease of these closely-related serovars. Of particular interest are the genes associated with the gastrointestinal-associated lifestyle of the broad host range serovars that have become pseudogenes in the host-specific Gallinarum and Pullorum strains. We also found evidence that the evolution of *Salmonella* towards host restriction occurs in sequential steps: first through the acquisition of new genes via horizontal gene of prophages and pathogenicity islands, followed by pseudogene formation and eventual gene loss.

## Methods

### Strains and culture conditions

*Salmonella enterica* Enteritidis LK5 was obtained from Dieter Schifferli, and *S*. *enterica* Dublin SARB12 and Pullorum RKS5078 were obtained from the *Salmonella* Genetic Stock Center, University of Calgary, Calgary, Canada. The strains were cultured in LB medium at 37° C for chromosomal DNA isolation. The ornithine decarboxylase assay was performed in Moeller decarboxylase broth supplemented with ornithine [25] and incubated at 37° C for 48 hours.

### Sequencing and bioinformatic analysis

Chromosomal DNA was isolated from overnight cultures using the Wizard Genomic DNA purification kit as described by the manufacturer (Promega U. S., Madison, WI, USA). The DNA was sequenced using both Sanger and 454 pyrosequencing methods. Pryosequencing was conducted by the Ecological Metagenomics Undergraduate Class at San Diego State University. Contigs were assembled from the reads using GS de Novo Assembler version 2.6 (454 Life Sciences, a Roche company, Branford, CT, USA) [26], and then scaffolded to publicly available reference genome sequences using blastn [27]. Enteritidis LK5 was scaffolded to the Enteritidis P125109 sequence (accession no. AM933172 [10]), and Dublin SARB12 was scaffolded to the Dublin CT02021853 sequence (accession no. CP001144 [28]) using scaffold_builder [29]. After the order of contigs around each chromosome was determined, draft genome sequences were assembled using a custom script with the gaps between contigs filled with N’s. Draft sequences were then aligned back to the respective reference genomes using the NUCmer module of MUMmer version 3.22 [30] to confirm correct assembly. The draft genomic sequences were then uploaded to and annotated by the RAST server [31], then visually inspected against the respective reference genomes using Artemis version 12.0 [32]. Putative single nucleotide polymorphisms (SNPs) and insertions or deletions (indels) were identified using the snpalign module of NUCmer/MUMmer [30] and validated using a custom script that analyzed the quality scores of the SNP base as well as the 10 bases flanking each side. SNPs were considered invalid if the quality score was <50 or if there was a flanking low score within a homopolymeric tract. Pseudogenes were validated with the Psi-Phi program [33] as well as performing whole genome alignments using progressiveMauve [34] (either the stand-alone version or the Geneious Pro 5.5.8 plug-in created by BioMatters available at http://www.geneious.com) to manually analyze and compare orthologous ORFs between sequences. All annotated pseudogenes in Dublin CT02021853 were scrutinized further by manually comparing their ORFs to the Typhimurium LT2 orthologue in the annotated genome sequence ([6]; accession no. NC_003197). Genes were considered pseudogenes if they were truncated more than 10% of the LT2 orthologue. Prophages were identified in the three Dublin genomes and the Pullorum genome using the PhiSpy program [35]. The sequences have been deposited in the European Molecular Biology Laboratory's European Nucleotide Archive (ENA) under the project ID PRJEB8699. The *S*. Enteritidis LK5 genome has the accession number ERS673772 and the *S*. Dublin SARB12 genome has the accession number ERS685404. In addition, both sequences are available from the RAST guest account (http://rast.nmpdr.org/; username guest; password guest) with accession numbers 272989.12 for LK5 and 98360.19 for SARB12.

### PCR assay to determine the chromosomal arrangement type of Enteritidis LK5 and Dublin SARB12

The PCR conditions used are described in [36]. The primer sequences and combinations used were the same as those described in [37].

## Results

### Chromosomal arrangement types of Enteritidis LK5 and Dublin SARB12

As seen in the Gallinarum 287/91 and Pullorum RKS5078 genomes [10,38], *Salmonella* strains belonging to host-specific serovars very often have large-scale chromosomal rearrangements from recombination between the seven *rrn* operons spread around the chromosome [39]. These rearrangements alter the chromosomal arrangement type, which is the order and orientation of the seven chromosomal regions between the *rrn* operons [40]. Broad host range *Salmonella* rarely have these types of rearrangements and typically have the “conserved” arrangement type. To properly scaffold the contigs, both the Enteritidis LK5 and Dublin SARB12 genomes were confirmed as having the “conserved” arrangement type using a PCR-based assay.

### Assembly of Enteritidis LK5 and Dublin SARB12 genome sequences

The 454 and Sanger reads of the Enteritidis LK5 and Dublin SARB12 genomes were assembled into 49 and 64 contigs respectively. Of these, 28 contigs of Enteritidis LK5 sequence were scaffolded around the Enteritidis P125109 genome, and 36 contigs of Dublin SARB12 sequence were scaffolded around the Dublin CT 02021853 genome. Unused contigs were either short and had multiple blastn hits, i.e. were present in multiple genomic copies, or were not present on the reference chromosome, i.e. the blastn hits were to plasmids. The location and size of gaps between the contigs were identified. In both Enteritidis LK5 and Dublin SARB12, less than 1.4% of referenced bases were gapped. In almost all cases, gaps were located in chromosomal regions present in multiple copies, for example *rrn* operons.

### Comparison of Enteritidis genomic sequences

Comparative analysis revealed 43 indels between the Enteritidis P125109 and LK5 genomes. Not counting prophage differences (see below), the indel size range of the 16 indels >1 bp was 3–232 bp with a mean of 39 bp (S1 Table). While two of these indels were intergenic, most of the others did not change the reading frame of the gene they were in. Only one indel caused a frameshift; an 11 bp deletion in LK5 occurred at the end of the *yjfK* gene, resulting in a fusion with the *yjfL* reading frame. The largest indel occurred in a cluster of tRNA-Gly genes; while three were present in P125109, only two were found in LK5. There were 26 indels identified between the P125109 and LK5 genomes that were 1 bp in size (S2 Table). Of these, 11 were intergenic, 2 restored the reading frame of identified P125109 pseudogenes SEN_0139 and *yegS*, and 12 truncated the LK5 gene product at least 10% relative to the P125109 homologue. In addition, 560 SNPs were identified between the P125109 and LK5 genomes (S3 Table). There were more than twice as many transitions than transversions. Almost half of these SNPs were non-synonymous, with six SNPs forming additional pseudogenes in LK5 by introducing premature stop codons and 7 SNPs correcting the reading frames of pseudogenes previously reported in P125109. One of these corrected genes was the virulence gene *ratB*.

### Comparison of Dublin genomic sequences

Comparison of the Dublin CT02021853 and SARB12 genomes revealed 79 indels. Of these, the 42 indels >1 bp averaged 219 bp and ranged from 2 bp to 4.4 kb in size (S4 Table). The largest indel is due to a duplication of the *gtr* operon in Dublin CT02021853 and is discussed in more detail below. Of the rest, 9 indels resulted in 9 pseudogenes in SARB12 and 1 pseudogene in CT02021853. In addition, two indels deleted 3 tRNA genes in SARB12 relative to CT02021853. The other 37 indels identified between the CT02021853 and SARB12 genomes were 1 bp in size (S5 Table). Of these, 15 were intergenic, 14 truncated the SARB12 gene product at least 10% relative to the CT02021853 orthologue, and 1 corrected the reading frame of CT02021853 identified pseudogene *yfbQ* (SeD_A2678 and SeD_A2679) in SARB12. Four 1 bp indels occurred in reading frames called as pseudogenes in both CT02021853 and SARB12, and the remaining three 1 bp indels did not appear to significantly truncate or alter the amino acid sequence of their residing reading frames. In addition to the indels, 632 SNPs were found between the CT02021853 and SARB12 genomes, with more than three times the number of transitions than transversions (S6 Table). Of these, 18% were intergenic and 32% were synonymous. Even though about half of the SNPs were non-synonymous, 19 introduced or changed a stop codon. Of these, five corrected the reading frames in SARB12 relative to CT02021853 and nine truncated the reading frames in SARB12 >10%.

In addition to the Dublin CT02021853/SARB12 comparison, the genome of Dublin SD3246 was also compared to CT02021853. These two genomes were found to be very similar. No indels were identified between the two strains; however the SD3246 genome sequence contained 28 gaps filled with N’s. These gaps averaged 15 bp and ranged from 1 to 90 bp in size and their effect on any reading frames they may be in was unknown. There were 594 SNPs identified between CT02021853 and SD3246 with the same high ratio of transitions to transversions as found between CT02021853 and SARB12 (S7 Table). Of these, 23% were intergenic and 32% synonymous. Of the 272 non-synonymous SNPs, 17 altered the reading frame where they resided; six restored their respective reading frames, with five having the same corrective SNPs as SARB12, and one SNP restored the reading frame of *garD*. The other 11 SNPs truncated the SD3246 reading frames >10%.

### Comparison of Gallinarum genomic sequences

The two serovar Gallinarum genomes analyzed in this study represent different biotypes, and were more diverse than the Enteritidis and Dublin genomes. Previous analyses have shown that these genomes possess different *rrn* arrangement types, large scale chromosomal rearrangements resulting from recombination between the *rrn* operons that are common in strains belonging to host-specific *Salmonella* serovars [10,38,39]. In addition to these rearrangements, these genomes also differed by 481 indels and 6,392 SNPs. The 184 indels >1 bp averaged 161 bp and ranged in size from 2 bp to 10.22 kb (S8 Table). The largest indel deleted 11 genes from the Pullorum genome. While four of these genes were annotated as pseudogenes in Gallinarum, other deleted genes encoded a putative lipoprotein, a putative oxidoreductase, two putative transcriptional regulators, a conserved hypothetical DNA binding protein, and three other hypothetical proteins. Another large deletion in the Pullorum genome (3.66 kb) contained most of the *tor* operon, which allows trimethylamine N-oxide to be used as a terminal electron acceptor (reviewed in [41]). Smaller Pullorum deletions occurred in 11 genes annotated as pseudogenes in Gallinarum, and at least 27 of these deletions were intergenic (17 more deletions occurred in genes annotated in the Gallinarum genome but not in the Pullorum genome). The largest deletion in the Gallinarum genome (1.33 kb) included the *mdt* operon that encodes a multi-drug transporter that also confers resistance to bile salts [42]. At least 25 of the Gallinarum deletions were intergenic; 8 occurred in genes annotated in the Pullorum genome but not in the Gallinarum genome, and 21 occurred in genes annotated in the Gallinarum genome but not in the Pullorum genome. Also, 25 Gallinarum deletions occurred within genes annotated as pseudogenes in the Gallinarum genome. Of the 297 1-bp indels, 132 were intergenic and 44 occurred within pseudogenes according to the Gallinarum annotation; 217 were intergenic according to the Pullorum annotation (S9 Table). There were 10X more SNPs identified between the Gallinarum and Pullorum genomes (S10 Table) than between the Enteritidis genomes and the Dublin genomes. The majority of these SNPs were transitions, with a ratio similar to that found between the Enteritidis genomes. Using the Gallinarum genome as the reference, 21% of the SNPs were classified as intergenic. Of these, 193 SNPs occurred within annotated genes in the Pullorum genome although 136 of these genes encoded hypothetical proteins. An additional 675 SNPs that occurred within annotated Gallinarum genes were classified as intergenic in the Pullorum genome. Of these annotated Gallinarum genes, at least 94 were pseudogenes and very often contained multiple SNPs. Approximately half of the SNPs were non-synonymous. While 9 of these SNPs removed a stop codon, 93 SNPs introduced a stop codon with 18 of these occurring in pseudogenes such as the virulence genes *ratB*, *sopA*, and *srfB* (part of this gene also underwent an inversion event); *emrB*, a multidrug resistance gene; and *sefC*, which encodes a fimbrial usher protein.

### Pseudogene content

The number of identified pseudogenes varied depending on how the genomes were annotated. While the Enteritidis P125109 was originally annotated with 113 pseudogenes, our analysis only found 111. The ORFs of 8 of these were corrected in the Enteritidis LK5 genome; however LK5 contained an additional 18 pseudogenes (S11 Table).

The reference Dublin CT02021853 genome was annotated with 289 putative pseudogenes, and the reference Dublin SD3246 genome was annotated with 133 pseudogenes. However, after comparing the orthologues of the CT02021853 “pseudogenes” with their Typhimurium LT2 orthologues, only 88 were confirmed to be pseudogenes with >10% of the ORF truncated (S12 Table). Furthermore, 55 CT02021853 “pseudogenes” did not have a LT2 orthologue. Of these, 29 encoded hypothetical proteins and 6 encoded transposases. The Dublin SARB12 and Dublin SD3246 genomes contained a similar number of confirmed pseudogenes, with the SD3246 genome having 86 and the SARB12 genome having 80 (S13 Table). Differences in pseudogene content were observed between the three Dublin strains with 8 CT02021853 confirmed pseudogenes corrected in SARB12 and 7 corrected in SD3246. The SD3246 genome contained 15 unique confirmed pseudogenes. The CT02021853 and SD3246 genomes contained a confirmed pseudogene (SeD_A3093 and SD3246_2993) that was full-length in SARB12 (SARB12_3045). The CT02021853 and SARB12 genomes contained one pseudogene (SeD_A0358 and SARB12_0352) that was full-length in SD3246 (SD3246_0348) as well as 9 confirmed pseudogenes that were not annotated in SD3246. The SD3246 and SARB12 genomes contained 4 confirmed pseudogenes that were full-length in CT02021853. Another 15 “pseudogenes” were deemed questionable based on their coding length relative to the LT2 orthologue or because they contained an internal TAG codon that most likely encodes for selenocysteine (S13 Table).

Our analysis only detected 246 of the 309 pseudogenes originally identified in the Gallinarum 287/91 genome (S14 Table) [10]. About 40% of the 63 originally identified pseudogenes not called by us as pseudogenes were either transposases or phage-related genes. Of the 246 Gallinarum 287/91 pseudogenes we identified, 95 overlapped with the 239 pseudogenes we found in the Pullorum RKS5078 genome (S14 Table). Some of these pseudogenes that may affect host-specificty and virulence include the fimbrial genes *stiC*, *stfF*, *stbC*, *sthA* and *sthE*, and the virulence genes *pqaA*, *sinH*, *sopA*, *sifB*, and *slrP*. We also found that *speC*, the gene encoding ornithine decarboxylase, is a pseudogene in both the Gallinarum and Pullorum genomes (SG_3008 and SPUL_3118). The biochemical activity of this enzyme has been used to distinguish between biotype Gallinarum and Pullorum strains as Pullorum strains are usually positive while Gallinarum strains are usually negative [43]. The inactiviation of *speC* in Pullorum RKS5078 was confirmed biochemically as this strain was negative for ornithine decarboxylase activity. The pseudogenes only present in the Pullorum RKS5078 genome include another virulence gene (*sifA*), genes involved in DNA repair (*polB*, *mutH*, and *mutL*), genes encoding thioredoxin-related proteins (*trxC* and *ybbN*), genes encoding enzymes involved in amino acid synthesis (*ilvG*, *ilvI*, and *trpE*), and genes involved in manganese and copper transport (*mntH* and *copA* respectively).

In addition, pseudogenes found in all three serovars occurred within the resident prophages and transposons, and encoded non-functional transposases, integrases, and proteins involved in phage tail assembly.

### Prophage content

The prophages present in the Enteritidis, Dublin, Gallinarum, and Pullorum genomes fell into seven classes (Table 1). The Enteritidis strains P125109 and LK5 vary in phage type as they possess different prophages within their genomes. Enteritidis P125109 belongs to PT4 and contains the ΦSE20 prophage as well as four other crytic prophages. While the genome of Enteritidis LK5 contains the same crytic prophages, this strain belongs to PT8, and instead of ΦSE20 contains ELPhiS, a Fels-2-like prophage integrated at a different chromosomal location within the tmRNA gene *ssrA* located in the homologous region between SEN_2612 and SEN_2613 of Enteritidis P125109 [44]. While these two prophages are presumably responsible for the phage types of Enteritidis P125109 and LK5, their role, if any, in the virulence differences seen between these two strains is unknown.

**Table 1:** Prophages present in Enteritidis, Dublin, Gallinarum, and Pullorum genomes.

Phage Class	Related Phage Group	Ent P125109	Ent LK5	Dub CT02021853	Dub SD3246	Dub SARB12	Gal 287/91	Pul RKS5078
1	ΦSE-1	Not present	Not present	ΦDub1	ΦDub1	ΦDub1	Not present	Not present
2	Gifsy-2	ΦSE10	ΦSE10	ΦDub2	ΦDub2	ΦDub2	Not present	Not present
3	Gifsy-2	ΦSE12	ΦSE12	ΦDub3	ΦDub3	ΦDub3	ΦGal1	ΦPul1
4	Gifsy-2	ΦSE12A	ΦSE12A	ΦDub3A	ΦDub3A	ΦDub3A	ΦGal1A	ΦPul1A
5	ΦST18	ΦSE14	ΦSE14	Not present	Not present	Not present	Not present	Not present
6	ΦST64B	ΦSE20	Not present	ΦDub4	ΦDub4	ΦDub4	Not present	Not present
7	Fels-2	Not present	ELPhiS	ΦDub5	ΦDub5	ΦDub5	Not present	Not present

**Fig 1:**
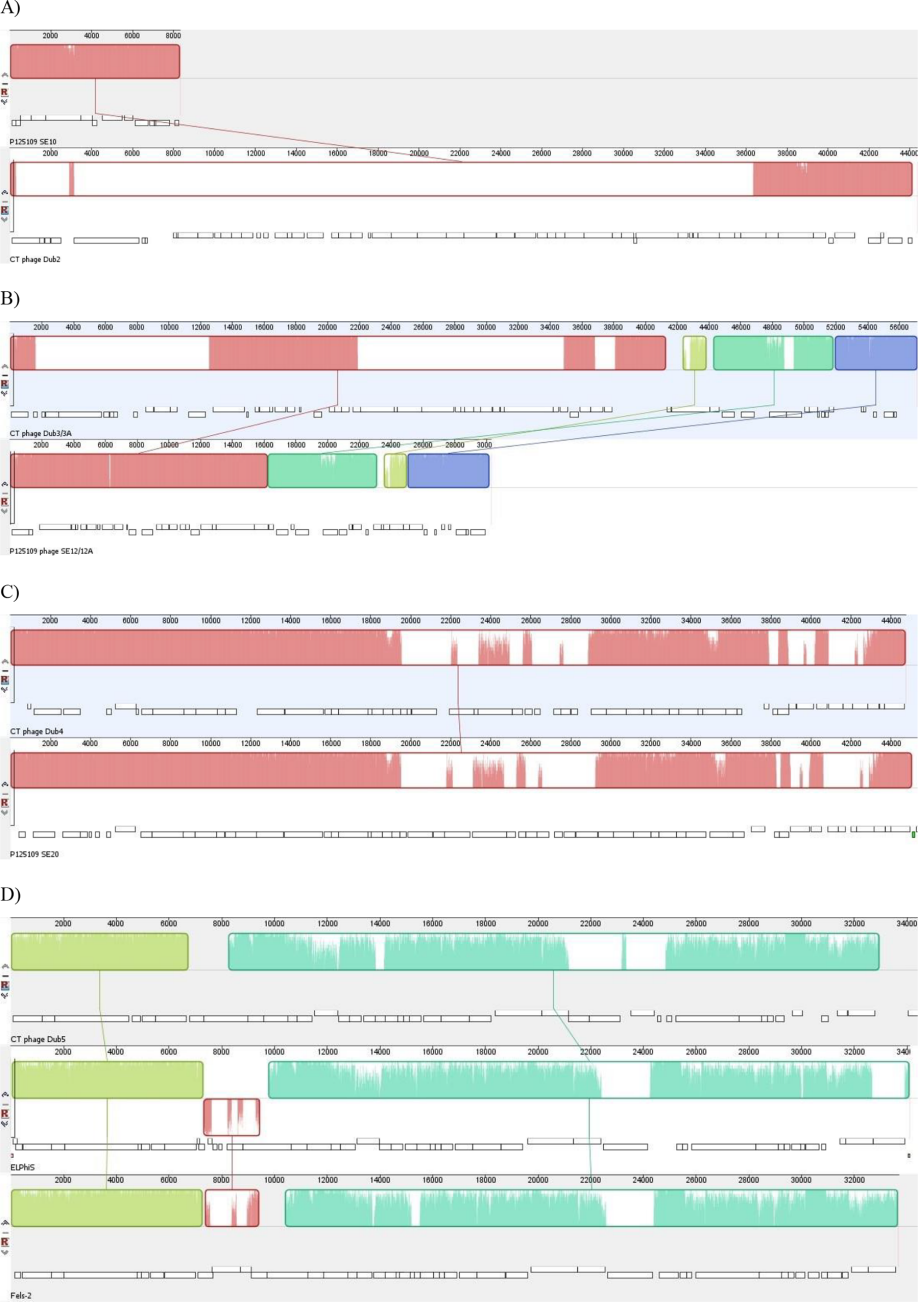
Mauve alignments of prophages present in the Enteritidis, Dublin, Gallinarum, and Pullorum genomes. A) Alignment of ɸSE10 and ɸDub2; B) Alignment of ɸDub3/3A and ɸSE12/12A; C) Alignment of ɸDub4 and ɸSE20; and D) Alignment of ɸDub5, ELPhiS and Fels-2.

In contrast, all three Dublin strains have the same repertoire of six prophages (Table 1). Four of these are similar to the Enteritidis P125109 prophages ΦSE10, ΦSE12/12A and ΦSE20 in sequence and integration sites, while the remaining two are similar to *Salmonella* phages SE1 [45] and Fels-2. The prophage similar to the SE1 phage, ΦDub1, integrated into the same tRNA-Arg gene where SPI-16 is in the Enteritidis strains. The *gtr* operon that defines SPI-16 is also present in these strains, as well as in SE1. This operon encodes genes involved in altering the surface O-antigen, and is duplicated in Dublin strains CT02021853 and SD3246, but not in SARB12. Dublin prophage ΦDub2 is a Gifsy-2-like prophage and has significant homology to ΦSE10 in shared sequence (99.5% identical at the nucleotide level). However an alignment of these two prophages showed that ΦSE10 has undergone two deletions that total almost 36 kb, whereas ΦDub2 appears to be relatively intact (Fig 1). Furthermore, while ΦDub2 contains the same virulence-contributing cargo genes *sseI*, *gtgE*, and *gtgF* (also annotated as *msgA*) found in ΦSE10 and Gifsy-2, ΦDub2 also has numerous genes not present in Gifsy-2 that encode hypothetical proteins. Dublin prophages ΦDub3 and ΦDub3A are also Gifsy-2-like prophages, but are less similar to Gifsy-2 than ΦDub2. Akin to ΦSE10 and ΦDub2, ΦSE12/12A has four deletions compared to ΦDub3/3A (Fig 1). One of the deletions includes the homologue of SeD_A1391, which encodes a diguanylate cyclase. Other deleted genes are involved in phage tail assembly and other phage functions. Furthermore, a 2 kb region also encoding phage tail assembly genes in ΦSE12A has translocated from ΦDub3A to ΦDub3. Dublin prophage ΦDub4 is a lambdoid-type prophage related to ΦST64B [46,47] and ΦSE20 in Enteritidis P125109 (Fig 1) [10], and all three prophages share the same tRNA-Ser integration site. Dublin prophage ΦDub5 is a P2-like prophage similar to the *Salmonella* phage Fels-2 and ELPhiS prophage found in Enteritidis LK5 (Fig 1), and shares the same *ssrA* integration site. As seen in a comparison between ELPhiS and Fels-2 [44], ΦDub5 differs in genes involved in tail assembly and cargo gene content. Six cargo genes were identified in ΦDub5; one encodes a putative lipoprotein whereas the other five encode hypothetical proteins, one of which is similar to gene 29c in ELPhiS.

The paucity of prophages in the genomes of serovar Gallinarum is in stark contrast to serovars Enteritidis and Dublin. Both the Gallinarum 287/91 and Pullorum RKS5078 genomes only contained the ΦSG12/12A [10] and ΦPul1/1A prophages respectively. These prophages are nearly identical to the ΦDub3/3A prophages, except for a 450 bp deletion in the ΦSG12 and ΦPul1orthologues of SeD_A1427 encoding a side tail fiber protein (SG_1231and SPUL_1707), and a 615 bp deletion in the ΦSG12A and ΦPul1A orthologues of SeD_A1432 and SeD_A1433 (encoding integrase and exodeoxyribonuclease 8). ΦDub3A also has a 1.8 kb deletion in the SG_1242 and SG_1243 orthologues encoding terminase and another tail fiber protein. ΦGal1A also has a 218 bp deletion within SG_1231 relative to ΦPul1A.

## Discussion

In this study we compared the genomic sequences of strains belonging to three serovars of *Salmonella enterica*: Enteritidis; Dublin; and Gallinarum. As these serovars vary in host-range and virulence but are closely related, a thorough comparison of genomes of different strains belonging to these serovars provides an excellent way to decipher the genomic differences responsible for the host-range and virulence of these serovars. We found that while the prophage content between the Enteritidis strains varied, the phage content of the Dublin strains was identical, as well as the phage content of the Gallinarum strains. Furthermore we identified pseudogene differences between strains belonging to the same serovar as well as between serovars. These pseudogenes were the result of numerous SNPs and indels that were found by comparing the genomes of strains belonging to the same serovar. Our results further illustrate two mechanisms known to play important roles in *Salmonella* genome evolution: 1) The acquisition of new genes via horizontal gene transfer (reviewed in [48]); and 2) The loss of gene function due to the accumulation of point mutations and indels that ultimately result in the formation of pseudogenes [7,10,49–52].

Previous studies have compared the genomes of serovars Enteritidis and Gallinarum, and the gene content of Dublin relative to Enteritidis and Gallinarum. Recently a genomic comparison of multiple strains belonging to Enteritidis and Dublin was published [21]. However this study failed to elaborate on differences in prophage content between these serovars or detect the *gtr* duplication in Dublin strains CT0202183 and SD3246. Furthermore, the study failed to validate the 289 annotated pseudogenes in CT02021853. While the results of these studies revealed genomic differences between these serovars, our study is the first to directly compare the genomic sequences of different strains belonging to all three serovars.

The annotations of the publicly available genomic sequences as well as our RAST-derived annotations for the Enteritidis LK5 and Dublin SARB12 genome sequences often differed in regards to the start codon used and whether or not a gene with inactivating mutations was split into two or more ORFs. Also, some ORFs present in one genome were not called in other genomes even though the sequences were identical. These annotation differences contributed to the differences in the number of genes annotated as pseudogenes, especially in the Dublin strains, and led us to reanalyze the called pseudogenes in all the genomes. While we found that almost all the annotated pseudogenes in the Enteritidis P125109 genome were confirmed, the number of annotated pseudogenes in the reference Dublin genomes (CT02021853 and SD3246) and the Gallinarum 287/91 genome [10] were substantially overestimated. One reason for this is that many pseudogenes were split into two or more annotated ORFs depending on the number of nonsense mutations and the length of the wild-type gene. We corrected for this in our analysis of the Dublin genomes by combining such ORFs into single pseudogenes.

The annotation differences we observed in this study have become a common problem as more genomes are sequenced and compared (reviewed in [53]). The variations in gene calling seen by us, for instance in open reading frames, different start sites and gene interruptions, when using different annotation programs have been analyzed by various groups, usually in the process of validating new bioinformatic tools for gene annotation [54–56]. Other approaches to gene annotation, for example using multiple genome alignments [57] and proteomics [58,59] can be used to improve the annotations of genomes. In our analysis we found that to properly identify pseudogenes in the Dublin SARB12 genome and to compare pseudogene content between Dublin genomes, the annotations of the called pseudogenes had to be manually compared to the annotated orthologues of a closely related genome of a strain known to not have a high pseudogene content, *S*. *enterica* sv. Typhimurium LT2 [6] as well as to each other to correct the overestimated number of pseudogenes in the publicly available genome sequences. The same approach was used to identify the pseudogenes in the Gallinarum 287/91 and Pullorum RKS5078 genomes.

Pseudogenes have been proposed to explain the differences in virulence and host specificity found between *Salmonella* serovars [7,50,52,60–62]. Differences in pseudogene content can also explain the variation in virulence observed between strains belonging to the same serovar. For example, the virulence gene *ratB* is a pseudogene in Enteritidis P125109 but not LK5, while another potential virulence gene, *mviM*, is a pseudogene in LK5 but not P125109. The three Dublin strains also varied slightly in pseudogene content; however no obvious differences in known virulence genes were found. The Gallinarum and Pullorum genomes contained several pseudogenes of virulence genes that are involved in intestinal colonization and intracellular survival in other animal models, suggesting that these functions are non-essential for infection of the fowl host.

Surface proteins, lipopolysaccharides, fimbriae, and flagella are often antigenic to the host’s immune system, and therefore under strong selective pressure, and consequently undergo antigenic variation. Many of the genes encoding these surface entities are also pseudogenes in the Gallinarum and Pullorum genomes. The Gallinarum serovar is known to be non-motile, in contrast to most other *Salmonella*, and both genomes contain pseudogenes that disrupt flagellar protein expression. In addition, many genes encoding fimbrial and other surface proteins are pseudogenes. These observations suggest that genes that encode for exposed proteins are being selected against by the host immune system during the process of host adaptation.

While pseudogenes were found in various metabolic pathways in all three serovars, the ability to anaerobically utilize propanediol and ethanolamine as carbon and energy sources was interrupted by multiple pseudogenes in the Gallinarum [10] and Pullorum genomes. Besides pseudogenes in the *eut* and *pdu* operons encoding the metabolizing enzymes for ethanolamine and propanediol respectively [63,64], pseudogenes were present in both the *cbi* operon required for cobalamin synthesis [65] and the *ttr* operon required for the anaerobic reduction of tetrathionate [66]. Both cobalamin and tetrathionate are required for anaerobic growth on ethanolamine and propanediol [66–68]. Cobalamin is produced endogenously under anaerobic conditions [69] and tetrathionate is produced by the oxidation of hydrogen sulfide present in the inflamed intestine [70]. Ethanolamine derived from intestinal host cell phosphatidylethanolamine has been shown to provide a growth advantage to *Salmonella* sv. Typhimurium in the inflamed mouse intestine [71] and promotes growth in contaminated food [72]. Propanediol is produced during the degradation of plant tissue and is thought to provide a nutritional source for *Salmonella* sv. Typhimurium outside its host [64,73] and during colonization of the chicken intestine [74]. The Gallinarum genome contained six pseudogenes in the *cob/cbi/pdu* region [10] versus one in Pullorum. The accumulation of pseudogenes in these operons is likely due to their coregulation by a positive regulatory protein encoded by *pocR* [75], which is a pseudogene in Gallinarum. As the genes in these operons are no longer being expressed and selected for, they appear to be deteriorating at a more rapid rate than the homologues in Pullorum. Overall, it appears that ethanolamine and propanediol utilization, as well as cobalamin synthesis in Gallinarum, is not necessary for avian-specific systemic infection, and may play a role in the adaptation to a host-specific lifestyle as these types of pseudogenes are also found in the human-specific Typhi serovar [7,8]. Our results from this analysis support those published previously by Nuccio and Bäumler who also showed that degradation of genes involved in vitamin B12 biosynthesis, tetrathionate respiration, DMSO respiration, TMAO respiration, etanolamine utilization and 1,2- propanediol utilization separate the different *Salmonella* serovars on the genomic level [76].

In addition to the pseudogenes in the *ttr* operon that prevent the anaerobic respiration of tetrathionate, the Gallinarum and Pullorum genomes also contained pseudogenes in the *dms* and *tor* operons that are required for the anaerobic respiration of dimethylsulfoxide (DMSO) and trimethyamine-N-oxide (TMAO), respectively [41]. While the role of anaerobic respiration in *Salmonella* pathogenesis and virulence is not well understood, using tetrathionate, DMSO and TMAO as terminal electron acceptors is not required by either serovar Gallinarum biotype for systemic infection in fowl. This may be due to lack of availability of these substances in the fowl host, and subsequent lack of selective pressure to maintain the ability to anaerobically respire them. The loss of the ability to respire anaerobically may also be an important step in the transition from a gastrointestinal lifestyle and the ability to infect several hosts to a systemic lifestyle in a specific host [77].

Another pseudogene of interest in the Pullorum RKS5078 genome was the *speC* gene encoding ornithine decarboxylase (SPUL_3118). The strain lacked ornithine decarboxylase activity and the *speC* pseudogene contained the same inactivating 4 bp deletion present in the Gallinarum 287/91 *speC* pseudogene, suggesting that RKS5078 is really a biotype Gallinarum strain. However, the RKS5078 genomic arrangement type was the most common Pullorum arrangement type, and contained a large-scale inversion from recombination between the *rrnD* and *rrnE* operons that is often found in biotype Pullorum genomes but rarely in biotype Gallinarum genomes [40]. The most parsimonious explanation is that the ancestor of RKS5078 acquired this inactivating mutation independently of biotype Gallinarum. As ornithine decarboxylase is used to biochemically distinguish biotypes Gallinarum and Pullorum, it would be interesting to determine the frequency of this mutation in the Pullorum population.

The prophage content found in the various Enteritidis, Dublin, and Gallinarum genomes reflects their evolutionary history. All the genomes contained two prophages at the same relative genomic position (ΦSE12/12A in Enteritidis, ΦDub3/3A in Dublin, ΦSG12/12A in Gallinarum, and ΦPul1/1A in Pullorum) that represent the most ancient lysogenization events, with the “A” prophages most likely integrating first. These prophages are the most degraded in the Enteritidis genomes due to the accumulation of pseudogenes and deletions, and are considered cryptic [10]. The Dublin genomes also shared an additional prophage (ΦDub2) with the Enteritidis genomes (ΦSE10). However, while ΦDub2 appears to be relatively intact, ΦSE10 is also cryptic due to large deletions. The Dublin genomes also contained two additional prophages similar to ones found in each of the Enteritidis genomes. In contrast, the Gallinarum and Pullorum genomes only contained the oldest prophages. Taken together these results suggest that the Gallinarum/Pullorum lineage diverged first from the most common ancestor after the most ancient lysogenization events, and the Enteritidis and Dublin lineages diverged after acquiring ΦSE10/ΦDub2, supporting previous findings that the Gallinarum/Pullorum lineage diverged first, followed by Enteritidis and Dublin lineages diverging [11]. The observation that the Gallinarum and Pullorum genomes are relatively free of prophages suggests that the fowl-specific *Salmonella* may be sensitive to more phages compared to Enteritidis and Dublin. Such phages would be useful in cocktails for use in prophylactic phage therapy, a rekindled approach to control *Salmonella* infections in poultry houses [78–80].

The increased number of prophages present in the Enteritidis and Dublin genomes could be a consequence of their lifestyle as mammalian pathogens. Prophages not only provide genes known to contribute to virulence and pathogenicity [81], for example *sopE*, but cargo genes with unknown functions, such as those present in the LK5 ELPhiS prophage [44] and Dublin ΦDub5, that may play important but unidentified roles. Genes encoding enzymes that alter the O-antigen are present in P22-like phages, such as the SE1 and ΦDub1, allowing for lysogenic conversion of the prophage host. While there is strong selective pressure for various O-antigen forms by interspecific gene transfer (reviewed in [82]), the duplicated *gtr* operon found in Dublin strains CT02021853 and SD3246 will allow for more O-antigen diversity in these strains as these operons evolve in a paralogous manner.

*S*. *enterica* as a species has evolved as a pathogen through a sequential order of events starting with the acquisition of genetic material by horizontal gene transfer, for example pathogenicity islands, and cargo genes on prophages and insertion elements. *Salmonella* evolution has continued through the acquisition of pseudogenes, which has also contributed to host adaptation of certain *Salmonella* serovars. Here we have shown how the genomes of strains belonging to three closely related *Salmonella* serovars have evolved by identifying these types of genomic differences, and how these differences contribute to host range and virulence. The Gallinarum and Pullorum genomes have undergone the most change in the form of pseudogene accumulation and large-scale chromosomal rearrangements, consequences of a host-specific, niche-restrictive lifestyle. The reduced selective pressure found in the exclusive niche of the specific host, as well as transmission bottlenecks and a small effective host population, allows for genetic drift and gene inactivation, as well as rearrangements to become fixed within the population [36,83]. In addition, the shift from a gut-associated lifestyle to a systemic lifestyle affects the selective pressure on the genes that contribute to life in the intestine. The Pullorum RKS5078 genome also contained pseudogenes in the mismatch repair genes *mutH* and *mutL*, which explains the high number of SNPs found between the Gallinarum 287/91 and Pullorum RKS5078 genomes. These results also suggest the Pullorum biotype is diversifying faster than biotype Gallinarum due to an increased accumulation of point mutations and pseudogenes. Hypermutable strains of pathogenic bacteria have been hypothesized to provide an advantage during host adaptation and colonization (reviewed in [84,85]) and mismatch deficient mutator strains are more susceptible to homeologous recombination (reviewed in [86]). The processes driving the evolution of these *Salmonella* serovars will be better understood as more genomes of strains belonging to these serovars are sequenced and analyzed.

Both Gallinarum/Pullorum and Dublin appear to be diseases of domestication. They are more similar to each other and to Enteritidis than *S*. Typhi is to is closest relatives, reflecting the time that each has had since separation of its host lineage. Humans separated from other primates ~5 million years ago, while cattle (*S*. Dublin) were domesticated ~10,000 years ago, and chickens domesticated ~8,000 years ago. Gallinarum/Pullorum and Dublin appear to have separated from their close relatives after these animals were domesticated, but presumably their spread has been aided by domestication including the selective breeding of animals and the removal of competing pathogens[17].

In summary, detailed genome comparisons of closely related *Salmonella* serovars provide insights into the tempo and mode of the evolution of host specificity. The process seems to be driven first by the acquisition of new genes by horizontal gene transfer, followed by pseudogene formation and loss of gene function during the colonization of new environmental niches.

## Supporting Information

S1 Table Indels greater than 1 bp between Enteritidis strains P125109 and LK5. (CSV)

S2 Table 1-bp Indels between Enteritidis strains P125109 and LK5. (CSV)

S3 Table Validated SNPs between Enteritidis strains P125109 and LK5. (CSV)

S4 Table Indels greater than 1 bp between Dublin strains CT02021853 and SARB12. (CSV)

S5 Table 1-bp Indels between Dublin strains CT02021853 and SARB12. (CSV)

S6 Table Validated SNPs between Dublin strains CT02021853 and SARB12. (CSV)

S7 Table SNPs between Dublin strains CT 02021853 and SD3246. (CSV)

S8 Table Indels greater than 1 bp between Gallinarum 287/91 and Pullorum RKS5078. (CSV)

S9 Table 1-bp Indels between Gallinarum 287/91 and Pullorum RKS5078. (CSV)

S10 Table SNPs between Gallinarum 287/91 and Pullorum RKS5078. (CSV)

S11 Table Serovar Enteritidis Pseudogenes (CSV)

S12 Table Serovar Dublin Pseudogenes (CSV)

S13 Table Serovar Dublin Questionable Pseudogenes. (CSV)

S14 Table Gallinarum 287/91 and Pullorum RKS5078 Pseudogenes. (CSV)
